# Administration of BNT162b2 mRNA COVID-19 vaccine to subjects with various allergic backgrounds

**DOI:** 10.3389/fimmu.2023.1172896

**Published:** 2023-08-15

**Authors:** Yaarit Ribak, Limor Rubin, Aviv Talmon, Zvi Dranitzki, Oded Shamriz, Isca Hershkowitz, Yuval Tal, Alon Y. Hershko

**Affiliations:** Allergy and Clinical Immunology Unit, Department of Medicine, Hadassah Medical Organization, Faculty of Medicine, Hebrew University of Jerusalem, Jerusalem, Israel

**Keywords:** COVID- 19, vaccination, drug allergy, anaphylaxis, polyethylene glycol

## Abstract

**Background:**

The mRNA-based COVID-19 vaccine was introduced to the general public in December 2020. Shortly thereafter, safety concerns were raised due to the reporting of allergic reactions. Allergy-related disorders were suspected to be significant risk factors and the excipient polyethylene glycol was suggested to be a robust allergen.

**Methods:**

This is a retrospective study analysis. Subjects with putative risk factors for severe allergic reactions to the Pfizer-BioNTech BNT162b2 vaccine were referred for vaccination under observation at the Unit of Allergy and Clinical Immunology. Data was collected for each subject, including demographic details, medical history and previous reactions to any allergen. When appropriate, skin tests were done prior to vaccination.

**Results:**

A total of 346 subjects received 623 vaccine doses under observation. The study included patients with various allergy-related disorders (n=290) and those with allergy to a previous COVID-19 vaccine dose (n=56). Both groups showed female predominance (78% and 88%, p=NS). Patients without reactions to previous doses reported more drug allergy (80% vs. 39%, p<0.001) and previous anaphylaxis (64% vs. 14%, p<0.001). There was no difference in sensitivity to other allergens, including polyethylene glycol. Under observation, mild allergic reactions were noted in 13 individuals characterized by female gender (100%), a history of anaphylaxis (69%) and drug allergy (62%). In 7 subjects, allergy was treated with antihistamines while others recovered spontaneously.

**Conclusion:**

Our study demonstrates that vaccination under specialist-supervision is a powerful tool for reducing over-diagnosis of systemic reactions and for rapid and reliable collection of vaccine safety data.

## Introduction

The rollout of COVID-19 vaccination in December 2020 was accompanied by concerns that the vaccine may be highly allergenic. This fear was prompted by several reports in the public media of individuals with a history of allergy who developed anaphylaxis immediately following injection. A high rate of allergic reactions to the mRNA-based vaccine was also suggested by clinical data (1) and polyethylene glycol (PEG) was suspected as a major culprit allergen (2, 3). PEG is present in a large variety of medications, cosmetic products and detergents and induces allergic reactions. A previous case series presented subjects who developed anaphylaxis to medications with excipients containing this compound (4). Therefore, it was suggested that, following vaccine injection, preexisting PEG-specific IgE activates mast cells and stimulates anaphylactic responses (5).

The uncertainty that accompanied the vaccination campaign rollout prompted the UK authorities to issue a statement that any individual with a medical history of anaphylactic responses to food, medications, or vaccines should not be vaccinated (6, 7). It was estimated that this decision would exclude 3-5% of the general population from the campaign due to self-reported severe allergic reactions to any allergen (7). This directive was revised three weeks later leaving only allergic reactions to vaccine components as the major contraindication (7). Concomitantly, experts suggested the existence of several risk factors including allergic sensitization to other vaccines, mastocytosis and severe asthma (8).

Consequently, frequent recommendation changes led to uncertainty regarding vaccine safety and to excessive reporting of allergic reactions (9) accompanied by both patient and physician hesitancy. The need for rapid collection of data regarding the risk for allergic reactions to the COVID-19 vaccine prompted the assembly of an intervention team at our hospital. This team constituted a framework for individuals whose putative risk factors would otherwise not allow them to be vaccinated.

## Methods

### Protocol for vaccination under observation

**Figure 1:**
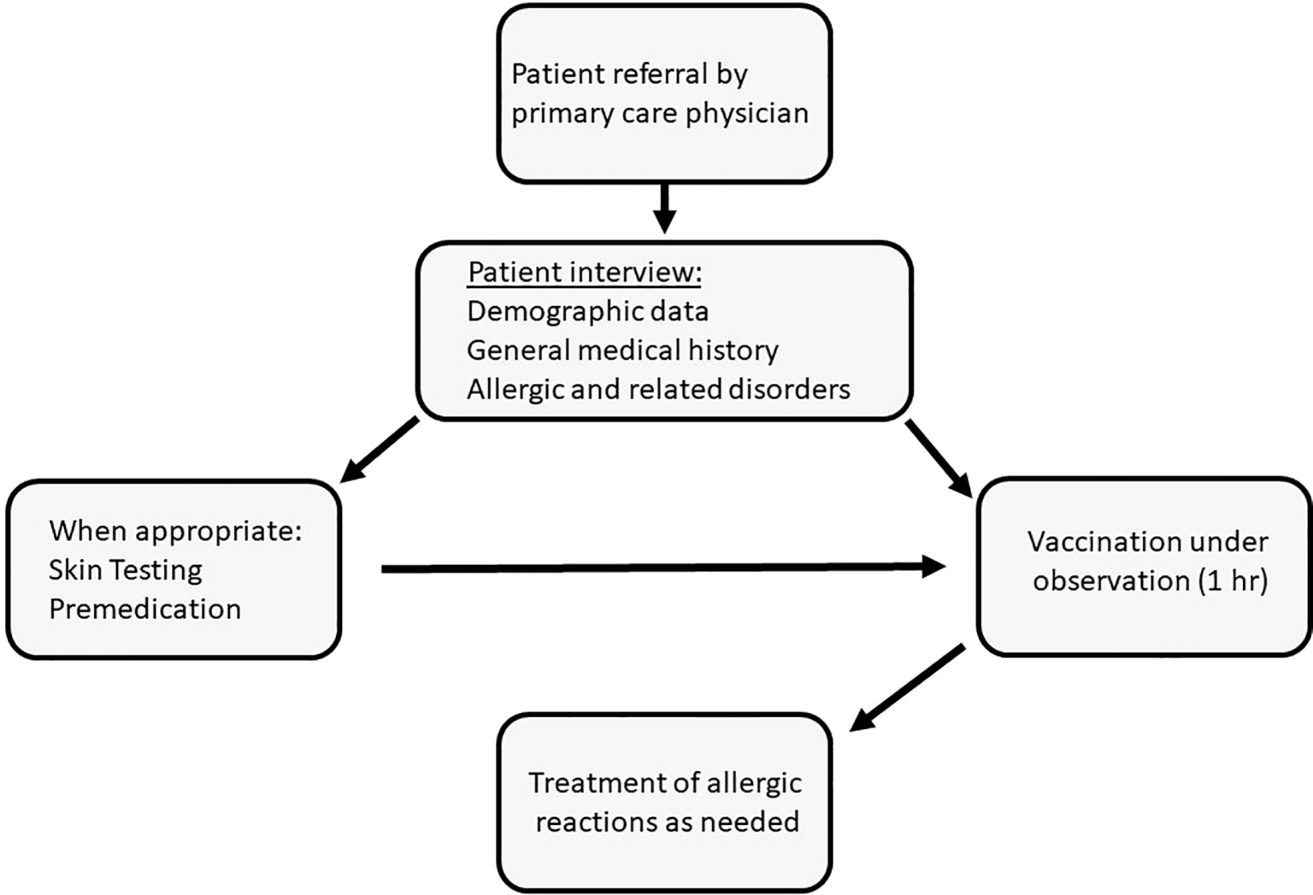
Vaccination under observation of subjects deemed to be at high risk for allergic reactions to the mRNA-based COVID-19 vaccine: algorithm of patient management.

This retrospective study presents the outcomes of a program for the vaccination of individuals with a history of allergy or related conditions that were considered as risk factors for anaphylaxis (**Figure 1**). These patients were referred from various regions of the country by their primary-care physicians to the Unit of Allergy and Clinical Immunology (UACI) from January to December 2021. Subjects were interviewed by the medical team for demographic data, medical history and details related to previous reactions to any allergen. Skin tests for the Pfizer-BioNTech BNTT162B2 and polyethylene glycol (PEG) were done for patients who had reported the following: immediate allergy to a previous COVID-19 vaccine dose; reactions to PEG-based laxatives; multi-drug allergies; sensitivity to relevant substances (e.g., detergents). Testing was also done in response to specific requests from the treating-clinician. Selected subjects received anti-histamines prior to vaccine injection and could receive injections in a graded manner. Premedication was administered to patients who reported an immediate reaction to a previous vaccine dose, subjects carrying an epinephrine autoinjector, and those with chronic spontaneous urticaria and angioedema or mastocytosis. Premedication was occasionally withheld despite these indications based on clinical judgement. All patients received Pfizer-BioNTech BNTT162B2, were observed for 1 hr and treated for reactions as needed.

### Statistical analysis

Continuous variables were compared by using student’s t-test. The comparison of proportions was performed by using Fisher’s exact test. Categorical variables with less than 5 observations in both groups were excluded from the analysis. The Bonferroni method was used for calculating adjusted p-value. Adjusted p-values<0.05 were considered significant.

The statistical analysis was performed with R (v. 4.0.2).

### Ethical considerations

Access to medical records for the purpose of this study was approved by the local Institutional Ethics Committee of Hadassah Medical Center in keeping with the principles of the Declaration of Helsinki (application number 0279-21-HMO). Due to the retrospective design of this study, no consent procedures were required.

## Results

### Characterization of study population

The study includes 346 subjects who were given 623 doses of COVID-19 vaccine under supervision (**Table 1**). Of these, 290 reported various allergy-related disorders while 56 had a history of allergy to a previous COVID-19 vaccine dose. The two categories showed a different mean age (57 ± 18 vs. 47 ± 17 years, respectively, p=0.005). Interestingly, both groups presented a robust female predominance (78% and 88%, respectively, p=NS). Each category was administered different proportions of vaccine doses 1, 2 and 3. Patients who did not have a history of reactions to previous doses had a higher rate of allergy to other drugs (80% vs. 39%, p<0.001) and previous episodes of anaphylaxis (64% vs. 14%, p<0.001). No difference was noted between the two groups with regards to previous allergic reactions to other specific allergens, including polyethylene glycol as well as other associated disorders, such as chronic urticaria or mastocytosis. However, patients with a putative COVID-19 vaccine allergy patients were more likely to receive anti-histamine premedication when vaccinated under observation (16% vs. 73%, p<0.001).

**Table 1:** Characterization of study population.

Characteristic				History of allergy to COVID-19 vaccine		
				No (n=290)	Yes (n=56)	Adjusted p-value (Bonferroni)
Age, yrs; mean ± S.D., range				57 ± 18, 14-89	47 ± 17, 7-84	0.005
Female gender; n (%)				226 (78)	49 (88)	NS
Vaccine dose administered during study; n (%)						
		1st		282 (98)	2* (5)	<0.001
		2nd		232 (80)	46 (80)	NS
		3rd		38 (13)	22 (39)	<0.001
History of allergy and related disorders; n (%)						
	Allergic reactions					
		Drug hypersensitivity		230 (80)	22 (39)	<0.001
		Food allergy		42 (15)	2 (4)	NS
		Insect venom allergy		18 (6)	2 (4)	NS
		Contrast media				
			gadolinium	5 (2)	0 (0)	NS
			Iodinated	12 (4)	1 (2)	NS
		Suspected PEG hypersensitivity**		10 (3)	4 (7)	NS
		Latex		8 (3)	1 (2)	NS
		Blood transfusion		2 (1)	0 (0)	NS
	Related conditions					
		Previous anaphylaxis		184 (64)	8 (14)	<0.001
		Epipen carrier		37 (13)	1 (2)	NS
		Chronic spontaneous urticaria or angioedema		29 (10)	4 (7)	NS
		Mastocytosis		6 (2)	0 (0)	NS
Antihistamine pre- medication, n (%)				46 (16)	41 (73)	<0.001

### Evaluation of reactions to prior COVID vaccine doses

In most cases, reactions to a previous vaccine dose occurred within 1 hour (59%) and were treated with antihistamines (n=14, 25%) followed by corticosteroids (n=10, 18%) and epinephrine (n=7, 13%) (**Table 2**). We evaluated the validity of these events as allergic reactions. Consequently, 1% of the events (n=2) was ruled out due to an onset time that exceeded 1 hour after injection. Conversely, 18% of the cases (n=10) were judged to be likely based on an immediate onset, involvement of more than 1 organ-system and at least one supporting objective finding. The remaining 80% (n=45) were immediate responses that were classified as unlikely since they did not meet the criteria for probable allergy.

**Table 2:** Characteristics of previously reported allergic reactions to COVID-19 vaccine.

Parameter		
Time of onset		
	< 30 min	24 (43)
	30 min - 1 hr	9 (16)
	N/A	23 (41)
Treatment; n (%)		
	Epinephrin	7 (13)
	Antihistamines	14 (25)
	Corticosteroids	10 (18)
Validation of allergic reactions; n (%)		
	Ruled-out	1 (2)
	Unlikely	45 (80)
	Likely	10 (18)

N/A, not available.,

### Allergic reactions to vaccination under observation at the Allergy Unit

Thirteen individuals reported an immediate response following the administration of COVID-19 vaccine under supervision during the study period (**Table 3**). Their mean age was 48 ± 13.6 (range 27-69) and, strikingly, they were all females. Most of these patients reported hypersensitivity to one or more drug (8/13, 62%) and a previous episode of anaphylaxis (9/13, 69%). The majority of this group had been administered anti-histamine premedication prior to vaccination (10/13, 77%). All allergic reactions were mild and 7 were treated with antihistamines. Objective findings were found in only 5 individuals (rash, local reaction, cough, rhinitis). The 3 patients who were suspected of having PEG allergy had mild rash and subjective sensations of tingling and swelling (patients 2, 4 and 8, respectively).

**Table 3:** Patients who reported allergic reactions under observation.

		History of allergy and related disorders						Vaccination under observation	
Patient	Age/Gender	Drug	Previous COVID-19 vaccine	PEG	Other	Anaphylaxis	Autoinjector	Antihistamine premedication	Reaction under observation
1	27/F	none	–	–	–	Yes	Yes	Yes	Throat tingling §
2	30/F	none	Yes	Yes	–	–	–	Yes	Redness and pruritus on chest
3	34/F	none	–	–	Chronic urticaria	–	–	Yes	Arm pruritus
4	39/F	>1	–	Yes	Chronic urticaria	Yes	–	Yes	Lip tingling
5	41/F	none	Yes	–	–	–	–	Yes	General tingling §
6	45/F	>1	–	–	Chronic urticaria	Yes	–	Yes	Local reaction §
7	49/F	>1	–	–	–	–	–	–	Multiple symptoms* §
8	49/F	>1	Yes	Yes	–	Yes	–	Yes	Swelling of face**
9	56/F	1	Yes	–	Insect	Yes	Yes	Yes	Cough
10	56/F	1	Yes	–	Insect	Yes	–	Yes	Pruritus, tongue swelling** §
11	62/F	>1	–	–	–	Yes	–	–	Rhinitis
12	67/F	none	–	–	–	Yes	Yes	Yes	Lip angioedema (late)
13	69/F	>1	–	–	–	Yes	–	–	Mouth tingling

*, Rash, dyspnea, shivering, elevated blood pressure and tachycardia, clear lungs; clinical impression of anxiety; **, no objective finding; §, treated with antihistamines.

## Discussion

This communication summarizes our experience in administering the Pfizer-BioNTech BNT162b2 to patients who were deemed to be at high risk for anaphylaxis. Several insights can be drawn from this study, which may be useful in clinical practice and in the understanding of challenges associated with future vaccination campaigns.

In line with previous studies, our work supports the conclusion that the Pfizer-BioNTech BNT162b2 is safe, and that PEG does not appear to be a significant allergen (9). It should be stressed that proven allergic sensitization to a specific component of a vaccine does not necessarily constitute a contra indication for vaccine administration. For example, it has been shown that children with egg allergy who receive egg-based influenza vaccine do not experience an increased rate of anaphylaxis (10). The Pfizer-BioNTech vaccine contains the putative allergen PEG whose capacity to induce sensitization corresponds to its molecular weight. Therefore, a reaction to a specific formulation of PEG does not necessarily predict sensitization to others (2). In our study, patients with a history of PEG allergy did not experience significant adverse events to the vaccine, and this may be attributed to its low molecular weight as an excipient, low injection volume and route of administration. Additionally, we have recently conducted a study on blood samples from 79 volunteers demonstrating an increase in PEG-specific IgG but not IgE, which was undetectable both before or after vaccine administration (11).

Furthermore, it has previously been shown that in subjects who report a reaction to the vaccine, subsequent doses are well-tolerated (12). Accordingly, we have previously demonstrated that the vast majority of reactions to the COVID-19 vaccine could not be validated as allergic, even when reporting had been done by healthcare workers (4). It has also been shown that reporting of allergy events was characterized by female gender and a self-reported history of allergy to other drugs (4). Interestingly, these two features were predominant in the present study as well, in which most subjects were referred due to anticipation of an allergic reaction while only a minority had already experienced a response to the vaccine. Intriguingly, subjects who were referred to our Unit following a previous response to the vaccine had a considerably low rate of reported allergy to other drugs, compared to the rest of the study population. Although this finding is not entirely understood, it could be at least partially explained by their younger age.

Analysis of immediate reactions that were observed during this study under our supervision may shed light on possible precipitating factors. All reactions were reported by female subjects and most of them had a history of allergy to other drugs and previous anaphylaxis. Although this may be partially explained by subjective complaints, it also raises the possibility of gender variances in response to drugs. These findings may be useful in predicting patients who are likely to develop immediate symptoms following vaccination.

This study has several limitations. First, the work was conducted in single center. However, this limitation is alleviated by the fact that patients were referred from various parts of the country and therefore the data was collected on a national level. Second, the retrospective design may entail an inherent bias. Nevertheless, data was collected by structured forms and we propose that this method of acquisition reduces the potential bias. Third, the number of patients who were recruited and the rate of allergic events compromise its statistical power. However, to the best of our knowledge, we present here the largest series of subjects thus far who were vaccinated safely despite guideline warnings. A related publication that was previously published (12) assessed the safety of vaccine administration to 18 subjects who had reacted to the first dose. In comparison, we report safety in 346 patients with a variety of putative risk factors and we show complete absence of significant systemic reactions. Consequently, we provide here comprehensive evidence to refute contraindications that were issued by leading health organizations.

In conclusion, our study highlights vaccination under specialist-observation as a powerful tool for allowing the administration of vaccine despite official contra-indications. This method can provide rapid support to hesitant individuals and their treating physicians as well as reliable data to policy leaders in crises such as an outbreak of a pandemic.

## Data availability statement

The raw data supporting the conclusions of this article will be made available by the authors, without undue reservation.

## Ethics statement

Access to medical records for the purpose of this study was approved by the local Institutional Ethics Committee of Hadassah Medical Center in keeping with the principles of the Declaration of Helsinki (application number In review 0279-21-HMO).

## Author contributions

YL, LR, and AT—contributed to study design, data collection, and wrote the first draft. ZD and OS—contributed to data collection. IH—organized and analyzed the data. YT and AH—contributed to conception, design and manuscript preparation. All authors contributed to manuscript revision, read, and approved the submitted version
